# Correlating *SFTPC* gene variants to interstitial lung disease in Egyptian children

**DOI:** 10.1186/s43141-022-00399-0

**Published:** 2022-08-08

**Authors:** Azza K. Abdel Megeid, Miral M. Refeat, Engy A. Ashaat, Ghada El-Kamah, Sonia A. El-Saiedi, Mona M. Elfalaki, Mona O. El Ruby, Khalda S. Amr

**Affiliations:** 1 Pediatric Department, Cairo University, Giza, Egypt; 2 Medical Molecular Genetics Department, Human Genetics and Genome Research Institute, National Research Centre, Cairo, Egypt; 3 Clinical Genetics Department, Human Genetics and Genome Research Institute, National Research Centre, Cairo, Egypt

**Keywords:** Childhood interstitial lung disease, Surfactant protein C, Variants, ILD genetics

## Abstract

**Background:**

Interstitial lung disease (ILD) is a broad heterogeneous group of lung disorders that is characterized by inflammation of the lungs. Surfactant dysfunction disorders are a rare form of ILD diseases that result from mutations in surfactant protein C gene (*SFTPC*) with prevalence of approximately 1/1.7 million births. *SFTPC* patients are presented with clinical manifestations of ILD ranging from fatal respiratory failure of newborn to chronic respiratory problems in children. In the current study, we aimed to investigate the spectrum of *SFTPC* genetic variants as well as the correlation of the *SFTPC* gene mutations with ILD disease in twenty unrelated Egyptian children with diffuse lung disease and suspected surfactant dysfunction using Sanger sequencing.

**Results:**

Sequencing of *SFTPC* gene revealed five variants: c.42+35G>A (IVS1+35G>A) (rs8192340) and c.43-21T>C (IVS1-21T>C) (rs13248346) in intron 1, c.436-8C>G (IVS4-8C>G) (rs2070687) in intron 4, c.413C>A p.T138N (rs4715) in exon 4, and c.557G>Ap.S186N (rs1124) in exon 5.

**Conclusion:**

The present study confirms the association of detecting variants of *SFTPC* with surfactant dysfunction disorders.

## Background

Interstitial lung diseases (ILDs) are a heterogeneous group of lung disorders, characterized by lung inflammation and fibrosis, which result in impaired gas exchange and in progressive cases lead to respiratory failure [1]. ILDs can be caused by systemic diseases or environmental factors with a worldwide incidence of 97/100,000 cases per year [2]. *SFTPC* mutations are inherited in an autosomal dominant pattern, and sporadic cases with de novo mutations may occur [3]. Lung damage caused by *SFTPC* mutations involves a “toxic gain of function” with accumulation of the misfolded protein within type 2 pneumocytes, with subsequent injury or apoptosis consequently ending in lung fibrosis [4]. Idiopathic pulmonary fibrosis (IPF) is the most common form of ILD in adults with worldwide incidence of approximately 20/100,000 males and 13/100,000 females [5], whereas surfactant dysfunction disorders represent rare forms of ILD in both children and adults with prevalence rate of approximately 1 in 1.7 million births [6]. Clinical diagnosis of ILD requires the presence of at least three of the following criteria in the absence of known primary disorders: respiratory symptoms as nonproductive dry cough, shortness of breath or intolerance of exercise, pulmonary signs such as tachypnea, retractions crackles, failure to thrive, or finger clubbing due to hypoxemia. Extrapulmonary manifestations such as joint involvement, skin rash, and systemic hypertension were detected in some patients [7]. Family history of similar conditions, and/or history of environmental exposure to chemicals, dusts, and birds, is an important predisposing factors [8]. Diagnosis is based upon a comprehensive history, a careful physical examination, and review of laboratory data, physiologic studies, radiography, and, in some cases, pathologic tissue obtained from lung biopsy. Multidisciplinary approach is essential for sound diagnostic and management decisions involving: radiology, surgery, pathology (lung biopsy), molecular biology, and physician assessment to reach the final diagnosis. Pulmonary function testing and bronchoscopy with bronchoalveolar lavage (BAL) are important investigations recommended to rule out pulmonary vascular disease. Echocardiography is important for detection of structural cardio vascular disease or pulmonary hypertension secondary to ILD which is of important prognostic implications [9].

Systemic diseases such as autoimmune diseases, or immune deficiency disorders, should be ruled out. Genetic studies can provide the final answer in diagnosis of ILD [10]. Surfactant dysfunction disorders are caused by mutations in one of three genes: surfactant protein B (*SFTPB*), surfactant protein C (*SFTPC*), and ATP-binding cassette member A3 (*ABCA3*), which are essential for the function and metabolism of pulmonary surfactant. *SFTPC* gene mutations are inherited in an autosomal dominant pattern, while mutations of *SFTPB* and *ABCA3* genes are inherited in an autosomal recessive pattern [11]. *SFTPC* gene, located on chromosome 8p21, encodes surfactant protein C (SP-C), a hydrophobic amino-acid polypeptide secreted into the alveolar space by alveolar type 2 epithelial cells to reduce surface tension [12]. ProSP-C domain plays an essential chaperone role in preventing the highly hydrophobic mature SP-C peptide from aggregating in the endoplasmic reticulum causing cell death [13]. More than 60 mutations in *SFTPC* gene have been identified in pediatric ILD patients, most of which are reported in patients of Caucasian or African descent with only few reports of Asian cases [14]. The second gene (*SFTPB*) is located on chromosome 2p11 and encodes surfactant protein B (SP-B), which is important for adsorption of secreted surfactant phospholipids to the alveolar surface [12]. The third gene is ATP-binding cassette transporter A3 (ABCA3) is expressed in type 2 alveolar epithelial cell lamellar bodies and plays an important role in pulmonary surfactant synthesis and transport [15]. There are nonspecific curative therapies for ILD, and only a few therapies might slow disease progression; however, management decisions would vary depending on ILD subtype [16]. Lung transplantation is possible for only urgent cases of idiopathic pulmonary fibrosis (IPF) patients [17]. The present study explores the association of *SFTPC* genetic variations with suspected surfactant dysfunction diseases in a cohort of Egyptian children.

## Methods

### Patients

Twenty unrelated ILD patients were enrolled in the present study after obtaining an informed consent from their guardians following the guidelines of the Institution Review Board. The included patients were referred from the outpatient allergy and pulmonology clinic, Abo El Reesh Hospital to the Clinical Genetics Clinic, Center of Excellence of Human Genetics. All patients were subjected to full history taking, family pedigree analysis, and thorough examination of all body systems with special emphasis on chest examination.

### Clinical criteria

The 20 studied probands were included based on presenting with breathing difficulties including the following: shortness of breath, coughing typically nonproductive, decreased exercise tolerance, fatigue, and/or weight loss. The excluded patients were those with an underlying cause of interstitial lung diseases such as immunodeficiency, collagen vascular disorders, environmental exposure, or gastroesophageal reflux disease (GERD).

### Molecular analysis

Genomic DNA was extracted from all 20 patients’ peripheral blood using Thermo Scientific GeneJET Genomic DNA Purification Kit (USA) according to manufacturer’s instructions. All the coding region and exon-intron boundaries were amplified with specific primers of *SFTPC* gene. The sequences of the designed primers are available on request. PCR cycling conditions were generated in (Perkin-Elmer Cycler, USA) as follows: 94 °C for 10 min followed by 35 cycles of 94 °C for 1 min, 59 °C for 1 min, and 72 °C for 1 min, followed by final cycles are extension at 72 °C for 10 min. The PCR products were purified using the ExoSAP Cleanup kit (Fermentas, Germany) and sequenced in both directions using the BigDye Terminator v3.1 Cycle Sequencing Kit (Applied Biosystems, Foster City, CA, USA) and analyzed on the ABI Prism 3500 Genetic Analyzer (Applied Biosystems, USA). Sequencing results were then analyzed on the NCBI website (http://blast.ncbi.nlm.nih.gov/Blast.cgi) and compared with cDNA sequence of *FTPC* gene (NM_003018.4).

## Results

### Clinical results

The twenty studied patients were descending from unrelated 20 pedigrees; positive consanguinity was detected in 11 out of the twenty included patients’ families.

Our studied cohort included 11 males and 9 females whose ages at examination ranged from 1 and 6/12 to 12 years. All patients were presenting with clinical criteria suggestive of ILD including cough and progressive dyspnea. Clubbing of fingers and toes was detected in elder patients. Short stature and failure to thrive were detected in 6/20 (30%) patients. Clinical data of all patients is summarized in Table 1.

**Table 1:** Summary of the clinical data of the patients

No.	Age	Sex	Consang.	Age of onset	Other affected member	Symptoms	Echo	Short stature/FTT	CT chest
P1	5 y	F	+ve	1 y	+ve	Cough-clubbing-dyspnea	−ve	++/−	Ground glass
P2	1.5 y	M	−ve	1 y	+ve	Cough-dyspnea	−ve	−/−	Ground glass
P3	7 y	M	+ve	1.5 y	+ve	Cough-cyanosis dyspnea-clubbing-OIH	PH	−/−	Ground glass
P4	8 y	M	+ve	2 y	−ve	Cough-dyspnea-clubbing	−ve	++/−	Ground glass
P5	19 y	F	+ve	4 y	−ve	Cough-dyspnea	PH	−/−	Mosaic
P6	10 y	F	−ve	1 y	−ve	Cough-dyspnea	PH	−/−	Ground glass
P7	6.5 y	M	−ve	2 y	−ve	Cough-dyspnea	PDA	−/++	Mosaic
P8	6 y	M	+ve	3 y	+ve	Cough-dyspnea-clubbing	PH	−/−	Ground glass
P9	11 y	F	−ve	2 y	+ve	Cough-dyspnea	−ve	−/−	Mosaic
P10	9 y	M	+ve	1.5 y	−ve	Cough-dyspnea-clubbing	PH	+/−	Ground glass
P11	11 y	F	+ve	2 y	+ve	Cough-dyspnea	PH-PFO	−/−	Ground glass
P12	8 y	F	−ve	1.5 y	+ve	Cough-dyspnea-clubbing	PH	−/−	Ground glass
P13	11 y	M	−ve	10 y	+ve	Cough-dyspnea-clubbing	−ve	−/−	Ground glass
P14	5 y	F	+ve	4 y	+ve	Cough-dyspnea-clubbing	−ve	−/−	Ground glass
P15	12 y	F	+ve	6 y	−ve	Cough-dyspnea-clubbing	+ve	++/−	Ground glass
P16	7 y	M	−ve	1 y	−ve	Cough-dyspnea-clubbing	−ve	−/−	Ground glass
P17	8 y	F	+ve	4 y	+ve	Cough-dyspnea	PH	−/−	Ground glass
P18	7 y	M	−ve	3 y	−ve	Cough-dyspnea	−ve	++/−	Ground glass
P19	10 y	M	+ve	5 y	+ve	Cough-dyspnea	PH	−/−	Ground glass
P20	8.5 y	M	−ve	2.5 y	−ve	Cough-dyspnea	−ve	−/−	Ground glass

*PH* pulmonary hypertension, *PDA* patent ductus arteriosus, *PFO* patent foramen oval, *FTT* failure to thrive

**Fig. 1:**
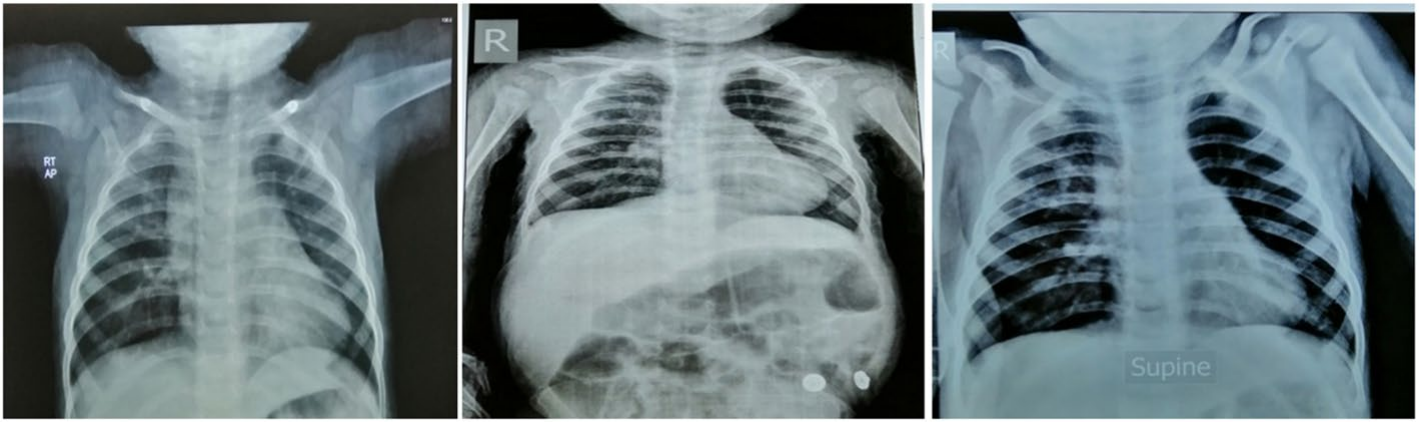
Plain chest radiographic examination showing patchy infiltrates in the lungs

**Fig. 2:**
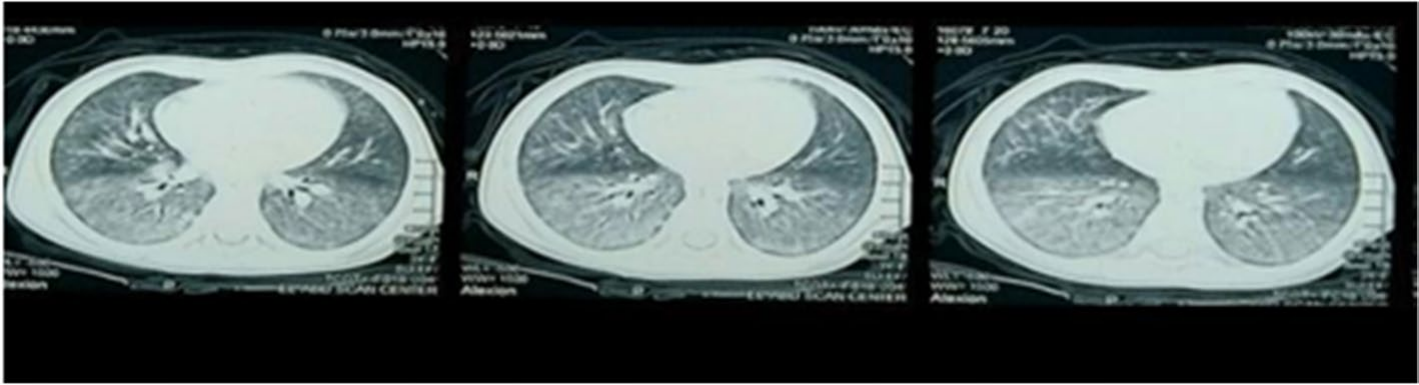
Bilateral lower lobar, to less extent middle lobar diffuse ground glass attenuation, reticulonodular densities, and peribronchial thickening (HRCT patient number 4)

**Fig. 3:**
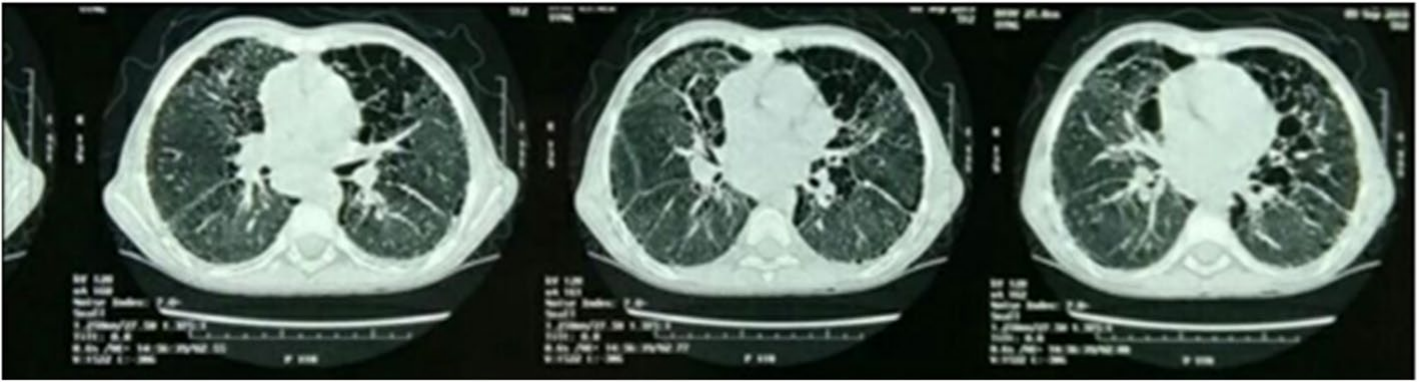
Ground glass appearance, peribronchial thickening, and cyst formation bilateral (HRCT patient number 5)

Plain chest radiographic examinations showed patchy infiltrates in the lungs of all the included patients (Fig. 1). High-resolution computed tomography (HRCT) revealed ground glass attenuation, honeycombing, and cyst formations. Ground glass attenuation opacities in CT chest were evident in our patients (Figs. 2 and 3). The echocardiographic evaluation detected pulmonary hypertension in 9 of the included patients and congenital heart diseases in the other 2 patients in the form of patent ductus arteriosus (PDA) and patent foramen oval (PFO).

### Results of molecular analysis

**Fig. 4:**
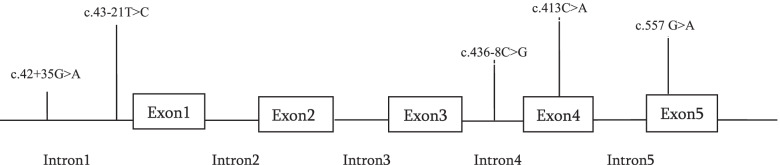
Schematic representation of localization of five reported variants in *SFTPC* gene: c.42+35G>A and c.43-21T>C in intron 1, c.436-8C>G in intron 4, c.413C>A in exon 4, and c.557G>A in exon 5

**Fig. 5:**
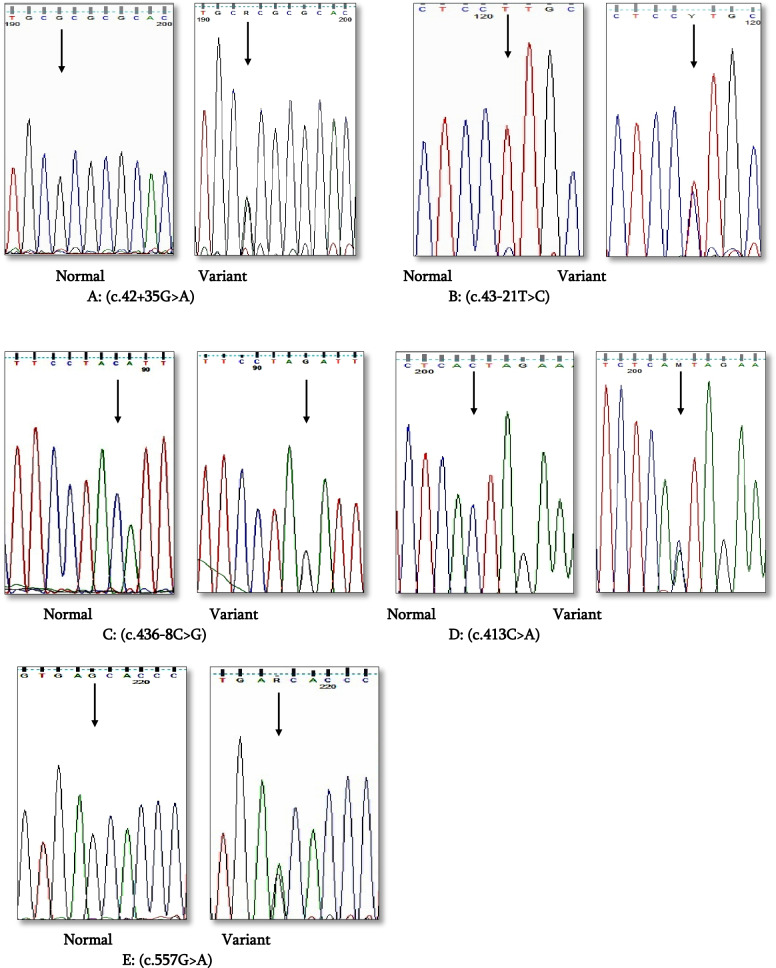
Sequence chromatograms showing five reported variants in *SFTPC* gene. **A** Normal and variant of (rs8192340) c.42+35G>A in intron 1, **B** normal and variant of (rs13248346) c.43-21T>C in intron 1, **C** normal and variant of (rs2070687) c.436-8C>G in intron 4, **D** normal and variant of (rs4715) c.413C>A in exon 4, and **E** normal and variant of (rs1124) c.557G>A in exon 5

Sanger sequencing of *SFTPC* gene of the twenty Egyptian ILD patients revealed five different variants in 17/20 unrelated patients: three intronic and two exonic variants. Three intronic sequence variants included the following: c.42+35G>A (IVS1+35G>A) (rs8192340) in one patient and c.43-21T>C (IVS1-21T>C) (rs13248346) in six patients, both variants were located in intron 1, while c.436-8C>G (IVS4-8C>G) (rs2070687) in intron 4 was detected in one patient. The two exonic missense variations included the following: c.413C>A (rs4715) in exon 4 disclosed from five patients resulting in the exchange of threonine by asparagine at codon 138 (p.T138N) and exon 5, c.557G>A (rs1124) gene variation, were detected in four patients resulting in substitution of serine with asparagine at codon 186 (p.S186N) (Figs. 4 and 5). No more variants were detected within the remaining three patients within *SFTPC* gene (Table 2).

**Table 2:** Variants identified in *SFTPC* gene

Patient number	Variant	Location	SNP	SIFT/polyphene
1, 2, 3	Negative			
4	c.42+35G>A (IVS1+35G>A)	Intron 1	rs8192340	Benign
5, 6, 7, 8, 9, 10	c.43-21T>C (IVS1-21T>C)	Intron 1	rs13248346	Benign
11	c.436-8C>G (IVS4-8C>G)	Intron 4	rs2070687	Benign
12, 13, 14, 15, 16	c.413 C>A (p.T138N)	Exon 4	rs4715	Possibly damaging
17, 18, 19, 20	c.557G>A (p.S186N)	Exon 5	rs1124	Benign

## Discussion

Interstitial lung diseases (ILDs) are a heterogeneous group of diseases characterized by widespread fibrotic and inflammatory abnormalities of the lung. Many forms of ILD are extremely rare, while other forms such as idiopathic pulmonary fibrosis (IPF) and sarcoidosis are seen commonly in general pulmonary practice. ILD refers to a heterogeneous collection of more than 200 distinct lung disorders that tend to be grouped together because they share clinical, radiographic, and pathologic features. These diseases have a broad spectrum of clinical presentations and manifestations ranging from lethal neonatal respiratory distress syndrome to adult chronic interstitial lung disease (ILD). In general, most interstitial lung diseases are characterized by four manifestations: (1) respiratory symptoms such as shortness of breath and cough, (2) specific chest radiographic abnormalities, (3) typical changes on pulmonary function tests in which the lung volume is decreased, and (4) characteristic microscopic patterns of inflammation and fibrosis [18–20].

Interstitial lung disease (ILD) in infants and children is associated with affected growth and high morbidity and mortality. All patients were recurrently admitted to hospital due to recurrent pneumonia, and they continued to have cough and tachypnea, and some of them exhibited failure to thrive later on [21].

Radiological investigations (chest X-ray) are the first approach as it is frequently abnormally diffused. But it has limited and low diagnostic specificity. Chest x-ray was performed for all studied 20 cases and showed patchy lung infiltrates. High-resolution computed tomography (HRCT), as a better diagnostic imaging technique for ILD children, helps avoid the need for surgical lung biopsy and is also useful in monitoring the therapeutic response. The most commonly observed findings in ILD by this technique are ground glass attenuation, honeycombing, and cyst formations. Ground glass attenuation opacities in CT chest were evident in all our patients and were our first clue for clinical diagnosis of ILD [22].

Echocardiography performed to the probands diagnosed 9 cases with pulmonary hypertension and 2 cases with congenital heart disease (patent ductus arteriosus (PDA) and patent foramen oval (PFO)); these findings were similar to a study, reporting a newly born female infant with neonatal pneumonia and PFO (1.9 mm). For some types of pediatric ILDs and few forms in adult ILDs, genetic causes have been identified and precise. Molecular diagnosis was used instead of the need for lung biopsies allowing definite diagnosis accessible to clinicians. Understanding the mechanisms of the idiopathic forms of interstitial lung disease is currently emerging. Studies of cells culture revealed a number of molecules and molecular pathways (such as transforming growth factor beta) that promote fibrosis [14, 23].

Previous studies reported genetic correlation of idiopathic pulmonary fibrosis (IPF) alterations in specific genes such as surfactant protein genes and telomerase. Further evidence for the role of genetics in interstitial lung disease comes from studies in patients with other disorders as sarcoidosis and Hermansky–Pudlak syndrome, where mutations in specific genes are associated with a higher incidence of lung fibrosis [24].

Egyptian study, conducted on 568 cases (191 males and 377 females) with mean age 44 ± 12 years from Upper Egypt, noted that IPF is the most common lung disease due to domestic air pollution, indoor exposures, and environmental factors [25].

Monoallelic mutations of the surfactant protein C gene (*SFTPC*) were associated with (ILD) in children and adults. When the SP-C gene is mutated, the precursor of surfactant protein C (proSP-C) is misfolded and accumulates within the ER and Golgi apparatus of AEC2s, leading to cellular injury and apoptosis [14].

In the present study, sequencing analysis of *SFTPC* gene within 20 ILD patients revealed five variants: c.42+35G>A (rs8192340), c.43-21T>C (rs13248346), c.436-26C>G (rs2070687), c.413C>A (rs4715), and c.557G>A (rs1124). A similar study was carried out on 760 Caucasian of Danish descents with different lung disease phenotypes as asthma, chronic obstructive pulmonary disease (COPD), and interstitial lung disease; they identified 18 variants including our five detected variants [26].

In patients with exonic mutations, the two previously reported *SFPTC* mutations in humans were described as acting in dominant fashion, with abrogation of SP-C synthesis (dominant negative effect). However, no deviation from the reference sequence was observed among patients harboring the variants predicted as benign after thorough *SFTPC* analysis, suggesting that either a cryptic *SFTPC* mutation segregated in this family, or that the *SP-C* deficiency was caused by a mutation at another genetic locus or by shared environmental exposures. Also, robust linkage disequilibrium was detected between the coding SNPs, T138N, and S186N by where the occurrences of the predictable haplotypes (for the allele frequencies of both SNPs) did not change considerably between patient and control groups. Remarkably, diverse histopathological types of pulmonary fibrosis were discovered in members of the same kindred harboring the same *SFTPC* mutation, and these diverse types were interpreted as possibly signifying pleiotropic expressions of the same genetic defect. *SFTPC*-associated familial forms of pulmonary fibrosis are characterized by reduced penetrance suggesting that further endogenous or exogenous elements might back the marked variety of pulmonary fibrosis predisposed by *SFTPC* mutations [13, 14, 27, 28].

In our study, single-nucleotide variants c.42+35G>A (rs8192340) and IVS4-8C>G (rs2070687), each was detected in two different patients (4 & 11); these variants were reported previously in 2 unrelated adult Dutch patients with familial pulmonary fibrosis though their mean ages at diagnosis were 54 years [29].

IVS4-8C>G (rs2070687) variant was reported in two American infants in two different studies; this variant was associated with persistent pulmonary hypertension (PPHN) and respiratory distress syndrome (RDS) symptoms [30, 31].

Sequencing analysis of (*SFTPC*) gene revealed two common missense variants p.T138N (rs4715) in exon 4 in five patients and S186N (rs1124) in exon 5 in four patients. p.T138N was predicted to be possibly damaging, affecting the domain of precursor SP-C (proSP-C) using in silico analysis, while the S186N variant was predicted to be polymorphism with no pathogenic effect [32].

The two common variants p.T138N and p.S186N in *SFTPC* gene were reported in two different studies, one in adult German (IPF) patients with age ranging from 24 to 61 years and the other in familial pulmonary fibrosis cases within population of Reunion Island, represented with unexplained respiratory distress (URD) [27, 28].

Moreover, the two variants p.T138N and p.S186N were described as minor alleles in two unrelated Finnish infants suffering respiratory distress syndrome (RDS) whose ages were less than 34 weeks with frequencies 0.20 and 0.22, respectively. Meanwhile, another study included 100 American patients assigned with familial pulmonary fibrosis, investigating the same two variants in frequencies of 0.255 and 0.318, respectively [33, 34].

Heterozygous *SFTPC* mutations concomitant, as well as heterozygous mutations in ATP-binding cassette transporter A3 (*ABCA3*) in infants with interstitial lung diseases (ILD), might likely led to development of clinical ILD [35].

Advances in understanding genetic factors contributing to ILD could outline some of the emerging roles of epigenetic modifications and give a vision about the progressed directions towards future prospects of genetically targeted therapies [18, 24].

Whole-exome sequencing (WES) has become an advanced approach to investigate rare alleles with direct functional consequences on protein products, affecting their pathways. WES was used to screen mutations concerned with surfactant proteins A and C (*SFTPA2* and *SFTPC*) genes; these two genes were mainly related to cell adhesion and immune response, which might partially explain changes of gene expression involved in immune-related pathways in ILD [36].

Molecular studies were performed using the next-generation sequencing of surfactant dysfunction genes identified three mutations in surfactant protein-C gene (*SFTPC*) in 6 Chinese children with ILD symptoms, whose ages of onset ranged from 7 days to 15 months: I73Tin 4/6, D105G in 1/6, and Y113H in 1/6 patients [37].

Our study highlights five variants: c.42+35G>A (IVS1+35G>A) (rs8192340) and c.43-21T>C (IVS1-21T>C) (rs13248346) in intron 1, c.436-8C>G (IVS4-8C>G) (rs2070687) in intron 4, c.413C>A p.T138N (rs4715) in exon 4, and c.557G>Ap.S186N (rs1124) in exon 5 within *SFTPC* gene that were all associated with interstitial lung disease (ILD) in Egyptian children.

## Conclusions

ILD comprises a heterogeneous group of diffuse parenchymal lung processes with overlapping clinical and histopathologic features. Progress has been made in identifying genes and pathways critical for ILD; the molecular and genetic causes of most lung malformations affecting lung function remain to be elucidated. Although the current study prides a diagnostic pilot seed, further studies should be carried out on a larger scale of Egyptian cases with symptoms of interstitial lung disease (ILD) to investigate all surfactant dysfunction causative genes (*SFTPA1*, *SFTPA2*, *SFTPB*, *SFTPD*, *ABCA3*, *TERT*, *TERC*, *TINF2*, *PARN*, *NAF1*, and *MUC5B*) by performing NGS-customized panel or whole-exome sequencing techniques. Advanced validation studies with larger statistical power are required to verify de novo findings and identify the underlying functional pathways. This will improve knowledge on the pathogenesis of associated surfactant diffuse lung disease in children and provide ILD families with a precise diagnosis as well as better genetic counseling.

## Data Availability

The data sets generated and/or analyzed during the current study are not publicly available due to patient’s privacy but are available from the corresponding author upon request.

## Abbreviations

*ABCA3*: ATP-binding cassette transporter A3
BAL: Bronchoalveolar lavage
COPD: Chronic obstructive pulmonary disease
FTT: Failure to thrive
GERD: Gastroesophageal reflux disease
HRCT: High-resolution computed tomography
ILD: Interstitial lung disease
IPF: Idiopathic pulmonary fibrosis
PDA: Patent ductus arteriosus
PFO: Patent foramen ovale
PH: Pulmonary hypertension
PPHN: Persistent pulmonary hypertension
RDS: Respiratory distress syndrome
SP-B: Surfactant protein B
SP-C: Surfactant protein C
*SFTPB*: Surfactant protein B gene
*SFTPC*: Surfactant protein C gene
