# Correction: Correlating *SFTPC* gene variants to interstitial lung disease in Egyptian children

**DOI:** 10.1186/s43141-022-00420-6

**Published:** 2022-09-21

**Authors:** Azza K. Abdel Megeid, Miral M. Refeat, Engy A. Ashaat, Ghada El-Kamah, Sonia A. El-Saiedi, Mona M. Elfalaki, Mona O. El Ruby, Khalda S. Amr

**Affiliations:** 1 Pediatric Department, Cairo University, Giza, Egypt; 2 Medical Molecular Genetics Department, Human Genetics and Genome Research Institute, National Research Centre, Cairo, Egypt; 3 Clinical Genetics Department, Human Genetics and Genome Research Institute, National Research Centre, Cairo, Egypt

**Correction: J Genet Eng Biotechnol 20, 117 (2022)**

**https://doi.org/10.1186/s43141-022-00399-0**

Following publication of the original article [1], the authors identified an error in the Authors’ contributions section for Engy A. Ashaat (EAA). The updated Authors’ contributions section is given below and the changes have been highlighted in **bold typeface**.

**Authors’ contributions**

AKA, **EAA**, and MME performed clinical selection, evaluation, and documentation of clinical criteria of the patients in the manuscript. MOR, SAES, and GEK interpreted clinical criteria and revised the manuscript. MMR performed the molecular and software analysis as well as wrote the manuscript. KSA analyzed and interpreted the results and revised the manuscript. The authors read and approved the final manuscript.

The Authors’ contributions section has been updated above and the original article [1] has been corrected.
